# Generalized Tremors in Dogs: 198 Cases (2003–2023)

**DOI:** 10.1111/jvim.70062

**Published:** 2025-03-28

**Authors:** Theofanis Liatis, Sofie F. M. Bhatti, Steven De Decker

**Affiliations:** ^1^ Department of Clinical Science and Services Royal Veterinary College Hatfield UK; ^2^ Small Animal Department Small Animal Teaching Hospital, Faculty of Veterinary Medicine, Ghent University Merelbeke Belgium

**Keywords:** corticosteroid‐responsive tremor syndrome, idiopathic cerebellitis, idiopathic generalized tremor syndrome, intoxication, mycotoxicosis, white shaker disease

## Abstract

**Background:**

Diseases associated with generalized tremors in dogs have not been extensively investigated in a large population of dogs.

**Hypothesis/Objectives:**

Describe semiology, tremor phenotype, and diseases associated with generalized tremors in dogs, and identify clinical features that could be associated with the underlying disease.

**Animals:**

A total of 198 dogs.

**Methods:**

Retrospective, single‐center study of dogs with generalized tremors and a final or presumptive diagnosis between January 2003 and December 2023.

**Results:**

The most common diseases associated with generalized tremors in dogs were intoxication (91/198; 46%), idiopathic generalized tremor syndrome (IGTS; 49/198; 24.7%), hypocalcemia (13/198; 6.6%), meningoencephalitis of unknown origin (MUO; 9/198; 4.5%), hypoglycemia (6/198; 3%), hypercalcemia (5/198; 2.5%) and degenerative encephalopathies (5/198; 2.5%). Dogs with IGTS were females (*p* = 0.002), younger (*p* = 0.002) with an acute progressive lateralizing presentation (*p* < 0.001 for all three), compared to dogs with intoxication that were males (*p* = 0.002), young and middle‐aged (*p* = 0.002) with a hyperacute nonprogressive symmetric presentation (*p* < 0.001 for all three). Behavioral changes (*p* = 0.01), hypersalivation (*p* = 0.04), abnormal mentation (*p* = 0.01), bilateral mydriasis (*p* = 0.02) or generalized hyperesthesia (*p* = 0.002) were common in intoxication, whereas hyporexia and vestibulocerebellar signs (*p* < 0.001 both) were common in IGTS. Dogs manifested only tremors in intoxication (45%) compared with IGTS (22%; *p* = 0.01). Improvement within 48 h from the onset of signs without corticosteroid treatment occurred exclusively in dogs with intoxication (*p* < 0.001).

**Conclusions and Clinical Importance:**

Intoxication and IGTS were the most common diseases associated with generalized tremors in dogs. Historical and clinical features may aid the clinician in adjusting their differential diagnoses and formulating a diagnostic and treatment plan.

**Abbreviations**
CSF: cerebrospinal fluid
CT: computed tomography
EMG: electromyography
FLAIR: fluid attenuation inversion recovery
FNIP2: folliculin‐interacting protein 2 gene
IGTS: idiopathic generalized tremor syndrome
IQR: interquartile ratio
MRI: magnetic resonance imaging
MUO: meningoencephalitis of unknown origin
PLP1: myelin proteolipid protein 1 gene
WEBLT: weight‐bearing lifting limb test

## 1 Introduction

Tremor is an involuntary, rhythmic, oscillatory movement of a body part [1]. Generalized tremors affect both thoracic and pelvic limbs [1]. Although the overall phenotype of generalized tremors is generally homogeneous (i.e., affecting the whole body), these tremors may have different features, such as being activated upon gravity (postural) or deteriorating with excitement and movement (action‐related kinetic), being episodic or continuous, or accompanied by intention head tremors [2]. Generalized tremor has been investigated previously in a cohort of 24 dogs in which idiopathic generalized tremor syndrome (IGTS) was the most common cause of tremors, followed by mycotoxin intoxication [3]. Other causes of generalized tremor in dogs also have been reported and include other toxic diseases (e.g., metaldehyde, avermectins) [4, 5], metabolic diseases (e.g., hypercalcemia, hypocalcemia, hyperphosphatemia, hyperchloremia, hypokalemia, hyperammonemia, hepatic encephalopathy) [6, 7, 8, 9], orthostatic tremor [10] and hypomyelination or dysmyelination [11].

Historically, the term tremor has been used inappropriately to describe not only tremors but also twitches, which may mimic tremors. For example, intoxications are characterized as tremorgenic throughout the literature. However, metabolic and toxic diseases can cause a variety of involuntary movements including tremors, fasciculations (peripheral nerve hyperexcitability), myoclonus, or epileptic seizures [2, 12, 13]. In some cases, generalized involuntary muscle contractions that are often erratic, with irregular frequency and variable amplitude, cause movement of the limbs while the dog retains usually consciousness and probably correspond to myoclonus rather than tremors. Nevertheless, the recognition of a tremor versus generalized myoclonus can be clinically challenging for the veterinarian. Thus, our study has adopted the word tremor to describe both true tremors and other related conditions.

To date, no recent study has investigated diseases associated with generalized tremor in a large population of dogs. Our aim was to investigate the causes of generalized tremor in dogs and identify any clinical features that could be associated with the underlying disease.

## 2 Materials and Methods

Our retrospective, single‐center, case‐series study was conducted in a referral hospital between January 2003 and December 2023. Because of the retrospective nature of the study, ethical approval was not required. Consent forms were signed by the owners of all dogs at admission, giving permission for the usage of video footage.

The digital medical database was searched for dogs that presented with generalized tremors. Search terms included: generalized or whole body or full body and tremor or twitch and dog or canine. Inclusion criteria consisted of (a) complete medical records, (b) presence or report of generalized tremor alone or accompanied by other clinical or neurological signs, and (c) a final or presumptive diagnosis. Exclusion criteria included focal tremors or tremors with an unclear description, cases without diagnosis, and other involuntary muscle contractions such as twitches as part of focal epileptic seizures, dystonic movements, or myoclonus.

Complete medical records consisted of clinical history, signalment, presenting complaints, physical and neurological findings, tremor phenotype, and final or presumptive diagnosis. Age was classified as young (< 3 years), middle‐aged (3–9 years) or old (> 9 years) [14]. Characterization of presentation included onset of neurological signs, progression, and lateralization [14]. Onset of signs was categorized into hyperacute (< 24 h), acute (1–7 days), subacute (7–15 days), and chronic (> 15 days) [10]. Progression of disease was set as progressive or nonprogressive [14]; progression of tremors was characterized by intensification or generalization of tremors (affecting all limbs with or without head, trunk or tail) and based on clinical history provided by owners or referring veterinarians at the time of initial presentation or clinical assessment during hospitalization [10]. Tremor phenotype included (a) periodicity (episodic, continuous), (b) intentional features (intentional, nonintentional) and (c) other features of tremors (e.g., deterioration with excitement) were recorded where available. All physical and neurological examinations were performed by a board‐certified neurologist or a neurology resident under the direct supervision of a board‐certified neurologist. Lateralization of the neurological signs (other than the tremors) was set as symmetric or asymmetric [14]. Video recording of tremors, hematology, serum biochemistry, abdominal ultrasound examination, computed tomography (CT), magnetic resonance imaging (MRI), cerebrospinal fluid (CSF) analysis, electromyography (EMG), motor nerve conduction studies, necropsy findings, and other diagnostic test results were evaluated when available. Magnetic resonance imaging was performed using a high‐field magnet (Intera 1.5T, Philips Healthcare, Amsterdam, Netherlands) and included transverse and sagittal T2‐weighted (T2W), transverse fluid attenuation inversion recovery (FLAIR), and transverse and sagittal T1‐weighted (T1W) pre‐ and post‐contrast (gadopentetate dimeglumine 0.1 mmol/kg IV bolus) images. Diagnosis was based on well‐established clinical, laboratory, and diagnostic imaging criteria for each disease category [2, 8, 15, 16, 17, 18].

Statistical analysis was performed using standard statistical software (SPSS Statistics 26, IBM Corporation, Armonk, New York). Data were assessed for normal distribution using the Shapiro–Wilk test for normality. Non‐normally distributed numerical variables are presented as median and range. Categorical variables were summarized as counts and percentages. Descriptive statistics were used for all cases. Univariate and multivariable analyses were performed only on diagnoses with > 30 available cases. Univariable analysis was performed using the Chi‐square test between the 2 most common diagnoses, intoxication and IGTS. Variables from signalment included breed, sex, neuter status, age, and body weight. Variables from history included onset of signs, progression of signs, symmetry of signs, tremor as the main complaint, lethargy, hyporexia, behavioral changes (including manic, running, aggressive, vacant, hallucinatory, or disoriented behavior), and gastrointestinal signs that included vomiting, diarrhea, hypersalivation, urinary incontinence, hyperthermia, and hypothermia. Variables from neurological findings included tremors being the only neurological finding, generalized hyperesthesia, abnormal mentation, abnormal posture including titubation, head tilt, wide‐based stance, abnormal gait (including ataxia), cranial nerve deficits (including menace response deficits), bilateral mydriasis, nystagmus, opsoclonus, blindness, neuroanatomical localization, and tremor phenotype (periodicity, additional intention head tremor, mandibular tremor, palpebral tremor, dancing sign). The dancing sign represented the shifting of weight from 1 limb to another presumably to avoid the sensation of the tremors [10]. Variables from outcome included improvement within 24–48 h from onset without corticosteroid administration. The relationship between demographic and clinical characteristics of dogs and diagnoses was first tested in the univariable analysis, and only variables with *p*‐values < 0.05 were entered into the multivariable analysis. Multivariable analysis was performed on all variables that reached significance in univariable analysis using logistic regression using the backward stepwise procedure. Significance level (*α*) was set at 0.05, and all statistical tests were 2‐tailed.

## 3 Results

Of the 572 cases that were found on search, 198 dogs met the inclusion criteria.

### 3.1 Final Diagnoses

Eighteen different causes were found in dogs with generalized tremors (Table 1). The most common disease categories associated with generalized tremors in dogs were toxic (95/198; 48%), inflammatory (59/198; 29.8%) and metabolic (33/198; 16.7%), with degenerative (5/198; 2.5%), idiopathic (4/198; 2%) and neoplastic (2/198; 1%) being uncommon. The most common final diagnoses included intoxication (91/198; 46%), IGTS (49/198; 24.7%), hypocalcemia (13/198; 6.6%), meningoencephalitis of unknown origin (MUO; 9/198; 4.5%), hypoglycemia (6/198; 3%), hypercalcemia (5/198; 2.5%) and degenerative encephalopathies (5/198; 2.5%). Causes of metabolic diseases were variable (Supporting Information S1).

**TABLE 1:** Final diagnoses in dogs with generalized tremors.

Final diagnosis	*n* (%)
Toxic causes (*n* = 91, 95.8%)	
Intoxication	91 (46)
Unknown agent	33/91 (36.3)
Mycotoxins	29/91 (31.9)
Metaldehyde	11/91 (12.1)
Avermectins	6/91 (6.6)
Cannabinoids	4/91 (4.4)
Mycotoxins and grapes	2/91 (2.2)
Chocolate (methylxanthines)	1/91 (1.1)
Plumb (cyanide)	1/91 (1.1)
Permethrins	1/91 (1.1)
Metronidazole	1/91 (1.1)
Drugs of abuse	1/91 (1.1)
Diet bar (xylitol/caffeine)	1/91 (1.1)
Tetanus	4 (2)
Inflammatory causes (*n* = 59, 29.8%)	
Idiopathic generalized tremor syndrome	49 (24.7)
Meningoencephalomyelitis of unknown origin	9 (4.5)
Acute idiopathic polyradiculoneuritis	1 (0.5)
Metabolic causes (*n* = 33, 6.7%)	
Hypocalcemia	13 (6.6)
Hypoglycemia	6 (3)
Hypercalcemia	5 (2.5)
Hepatic encephalopathy—portosystemic shunts	4 (2)
Diabetic polyneuropathy	1 (0.5)
Hypernatremia	1 (0.5)
Hyperammonemia	1 (0.5)
Hypoadrenocorticism	1 (0.5)
Suspected hypercatecholemia (pheochromocytoma)	1 (0.5)
Degenerative causes (*n* = 5, 2.5%)	
Spinocerebellar ataxia (potassium inwardly rectifying channel subfamily J member 10 [KCNJ10] gene mutation)	1 (0.5)
Subacute necrotizing encephalopathy (with moderate demyelination; histopathologically confirmed)	1 (0.5)
Suspected lysosomal storage disease	1 (0.5)
Suspected cerebellar cortical degeneration	1 (0.5)
Suspected mitochondrial encephalopathy	1 (0.5)
Idiopathic causes (*n* = 4, 2%)	
Primary orthostatic tremor	2 (1)
Benign idiopathic rapid postural tremor	2 (1)
Neoplastic causes (*n* = 2, 1%)	
Central nervous system lymphoma	1 (0.5)
Pituitary mass	1 (0.5)

### 3.2 Epidemiology

Most dogs with generalized tremors were purebreds (145/198; 73.2%). One hundred three dogs (52%) were females, and 95/198 (48%) were males, of which a total of 133/198 (67.2%) were neutered. Median age at onset of the tremors was 3.2 years (range, 3 months–14.3 years; interquartile range [IQR], 1.3–7.6 years), with 94/198 (47.5%) of dogs being young, 71/198 (35.9%) middle‐aged, and 33/198 (16.7%) old. Median body weight was 10.9 kg (range, 1.29–53 kg; IQR, 7.4–20 kg). The most commonly represented breeds were crossbreeds (49/198; 24.7%), Labrador retrievers (22/198; 11.1%), Jack Russell terriers (19/198; 9.6%) and West Highland White terriers (14/198; 7.1%; Supporting Information S2). Onset of neurological signs was hyperacute (97/198; 49%), acute (61/198; 30.8%) or chronic (40/198; 20.2%); no dog presented with subacute onset. Neurological signs were progressive (153/198; 77.3%) or nonprogressive (45/198; 22.7%), and symmetric (174/198; 87.9%) or lateralized (24/198; 12.1%). Generalized tremors were the main reason for referral in 84/198 (42.4%) dogs.

Signalment, presentation, clinical and neurological findings, diagnostic findings, treatment, and outcome for the most common diagnoses (*n* > 4) in dogs with generalized tremors are presented in Table 2.

**TABLE 2:** Signalment, presentation, clinical and neurological findings, diagnostic test results, treatment and outcome in the most common diagnoses (*n* > 4) in dogs with generalized tremors.

	Intoxication	IGTS	Hypocalcemia	MUO	Hypoglycemia	Hypercalcemia	Degenerative encephalopathy
*n* (%)	91/198 (46)	49/198 (24.7)	13/198 (6.6)	9/198 (4.5)	6/198 (3)	5/198 (2.5)	5/198 (2.5)
Signalment							
Median age (range; IQR [years])	3.4 (0.25–14.00; 5.3)	2 (0.3–10.0; 1.9)	4 (0.25–12.3; 9.35)	2.9 (0.75–11.6; 6.95)	8.85 (7–14.3; 6.1)	9.2 (8–12.5; 3.15)	6.1 (0.25–9.9; 8.78)
Sex (%)							
Female	40/91 (44)	35/49 (71.4)	10/13 (76.9)	5/9 (55.6)	3/6 (50)	3/5 (60)	1/5 (20)
Male	51/91 (56)	14/49 (28.6)	3/13 (23.1)	4/9 (44.4)	3/6 (50)	2/5 (40)	4/5 (80)
Neutered (%)	63/91 (69.2)	32/49 (65.3)	6/13 (46.2)	3/9 (66.6)	6/6 (100)	4/5 (80)	3/5 (60)
Breed (%)							
Purebred	69/91 (75.8)	31/49 (63.3)	11/13 (84.6)	7/9 (77.8)	5/6 (83.3)	5/5 (100)	3/5 (60)
Crossbreed	22/91 (24.2)	18/49 (36.7)	2/13 (15.4)	2/9 (22.7)	1/6 (16.7)	0/5 (0)	2/5 (40)
Median body weight (range; IQR [kg])	11.95 (2.5–36.5; 14.51)	10.6 (4.35–35.5; 7.8)	8.75 (4–38.8; 8.66)	5.98 (1.6–19.3; 5.44)	9.4 (8.6–27.1)	32.6 (10.3–37.7; 22.05)	6.92 (1.29–9.10)
Clinical signs							
Lethargy	13/91 (14.3)	11/49 (22.4)	6/13 (46.2)	5/9 (55.6)	5/6 (83.3)	4/5 (80)	0 (0)
Hyporexia	1/91 (1.1)	11/49 (22.4)	5/13 (38.3)	3/9 (33.3)	0 (0)	5/5 (100)	0 (0)
GI signs	38/91 (41.8)	18/49 (36.7)	6/13 (46.2)	2/9 (22.2)	0 (0)	1/5 (20)	0 (0)
Vomiting	34/91 (37.4)	17/49 (34.7)	5/13 (38.5)	2/9 (22.2)	0 (0)	1/5 (20)	0 (0)
Diarrhea	8/91 (8.8)	5/49 (10.2)	2/13 (15.4)	0 (0)	0 (0)	0 (0)	0 (0)
Hyperthermia	17/91 (18.7)	14/49 (28.6)	4/13 (30.8)	3/9 (33.3)	0 (0)	0 (0)	1/5 (20)
Urinary incontinence	4/91 (4.4)	0 (0)	0 (0)	0 (0)	0 (0)	0 (0)	1/5 (20)
Generalized hyperesthesia	29/91 (31.9)	4/49 (8.2)	2/13 (15.4)	1/9 (11.1)	0 (0)	0 (0)	1/5 (20)
Presentation							
Onset							
Hyperacute	90/91 (98.9)	0 (0)	5/13 (38.5)	1/9 (11.1)	1/6 (16.7)	0 (0)	0 (0)
Acute	0 (0)	38/49 (77.6)	4/13 (30.8)	3/9 (33.3)	0 (0)	4/5 (80)	2/5 (40)
Chronic	1/91 (1.1)	11/49 (22.4)	4/13 (30.8)	5/9 (55.6)	5/6 (83.3)	1/5 (20)	3/5 (60)
Progression							
Progressive	48/91 (52.7)	49/49 (100)	13/13 (100)	9/9 (100)	4/6 (66.7)	5/5 (100)	5/5 (100)
Nonprogressive	43/91 (47.3)	0 (0)	0 (00)	0 (0)	2/6 (33.3)	0 (0)	0 (0)
Lateralization							
Symmetric	89/91 (97.8)	35/49 (71.8)	13/13 (100)	3/9 (33.3)	6/6 (100)	5/5 (100)	5/5 (100)
Lateralized	2/91 (2.2)	14/49 (28.6)	0 (0)	6/9 (66.7)	0 (0)	0 (0)	0 (0)
Accompanying neurological deficits other than tremor	50/91 (54.9)	38/49 (77.6)	5/13 (38.5)	8/9 (88.9)	4/6 (66.7)	0 (0)	5/5 (100)
Neurologic findings							
Abnormal mentation	21/91 (23.1)	3/49 (6.1)	3/13 (23.1)	3/9 (33.3)	3/6 (50)	0 (0)	1/5 (20)
Titubation	1/91 (1.1)	8/49 (16.3)	0 (0)	2/9 (22.2)	0 (0)	0 (0)	0 (0)
Head tilt	0 (0)	9/49 (18.4)	0 (0)	5/9 (55.6)	0 (0)	0 (0)	0 (0)
Tetra‐ataxia	30/91 (33)	35/49 (71.4)	0 (0)	8/9 (88.9)	0 (0)	0 (0)	3/5 (60)
CN deficits	22/91 (24.2)	32/49 (65.3)	0 (0)	5/9 (55.6)	1/6 (16.7)	0 (0)	4/5 (80)
Menace response deficits	11/91 (12.1)	21/49 (42.9)	0 (0)	4/9 (44.4)	1/6 (16.7)	0 (0)	3/5 (60)
Opsoclonus	4/91 (4.4)	11/49 (22.4)	0 (0)	1/9 (11.1)	0 (0)	0 (0)	0 (0)
Nystagmus	0 (0)	10/49 (20.4)	0 (0)	2/9 (22.2)	0 (0)	0 (0)	1/5 (20)
Neuroanatomical localization							
Forebrain	0 (0)	0 (0)	0 (0)	0 (0)	0 (0)	0 (0)	1/5 (20)
Cerebellar	7/91 (7.7)	14/49 (28.6)	0 (0)	0 (0)	0 (0)	0 (0)	1/5 (20)
Vestibulocerebellar	7/91 (7.7)	25/49 (51)	0 (0)	0 (0)	0 (0)	0 (0)	1/5 (20)
Multifocal	42/91 (46.2)	5/49 (10.2)	4/13 (30.8)	9/9 (100)	4/6 (66.7)	0 (0)	1/5 (20)
Tremors only	35/91 (38.5)	5/49 (10.2)	9/13 (69.2)	0 (0)	2/6 (33.3)	5/5 (100)	0 (0)
C1‐5 SCS	0 (0)	0 (0)	0 (0)	0 (0)	0 (0)	0 (0)	1/5 (20)
Diagnostic testing							
MRI scan							
Performed	1/91 (1.1)	46/49 (93.9)	1/13 (7.7)	9/9 (100)	0 (0)	0 (0)	5/5 (100)
Normal	1/1 (100)	46/46 (100)	1/1 (100)	1/9 (11.1)	0 (0)	0 (0)	1/5 (20)
Abnormal	0 (0)	0 (0)	0 (0)	8/9 (88.9)	0 (0)	0 (0)	4/5 (80)
CSF analysis							
Performed	1/91 (1.1)	43/49 (87.8)	0 (0)	8/9 (88.9)	0 (0)	0 (0)	5/5 (100)
Normal	1/1 (100)	10/43 (23.3)	0 (0)	2/8 (25)	0 (0)	0 (0)	5/5 (100)
LP	0 (0)	22/33 (66.7)	0 (0)	2/8 (25)	0 (0)	0 (0)	0 (0)
MNP	0 (0)	11/33 (33.3)	0 (0)	2/8 (25)	0 (0)	0 (0)	0 (0)
MCP	0 (0)	0 (0)	0 (0)	2/8 (25)	0 (0)	0 (0)	0 (0)
Treatment							
Treatment							
Yes	90/91 (98.9)	49/49 (100)	10/13 (76.9)	9/9 (100)	5/6 (83.3)	3/5 (60)	3/5 (60)
No	1/91 (1.1)	0 (0)	3/13 (23.1)	0 (0)	1/6 (16.7)	2/5 (40)	2/5 (40)
Improvement within 24–48 h without corticosteroids	91/91 (100)	0 (0)	13/13 (100)	0 (0)	6/6 (100)	5/5 (100)	0 (0)
Outcome							
Follow‐up on discharge as per tremors							
Euthanasia	2/91 (2.2)	0 (0)	1/8 (12.5)	0 (0)	1/6 (16.7)	2/4 (50)	0 (0)
Deterioration	0 (0)	1/48 (2.1)	0 (0)	0 (0)	0 (0)	0 (0)	2/5 (40)
Static	0 (0)	1/48 (2.1)	0 (0)	1/9 (11.1)	0 (0)	1/4 (25)	2/5 (40)
Improved	89/91 (97.8)	46/48 (95.8)	7/8 (87.5)	8/9 (88.9)	5/6 (83.3)	1/4 (25)	0 (0)
		*1 missing	*5 missing			*1 missing	

Abbreviations: CN, cranial nerve; CSF, cerebrospinal fluid analysis; GI, gastrointestinal; IGTS, idiopathic generalized tremor syndrome; LP, lymphocytic pleocytosis; MCP, mixed cell pleocytosis; MNP, mononuclear pleocytosis; MRI, magnetic resonance imaging; MUO, meningoencephalomyelitis of unknown origin; SCS, spinal cord segments.

### 3.3 Tremor Phenotype

Generalized tremors were present in all dogs (Video 1). They were usually continuous (173/198; 87.4%) rather than episodic (25/198; 12.6%). In a few dogs, additional intention head tremor (8/198; 4%), palpebral tremor (7/198; 3.5%) or mandibular tremor (5/198; 2.5%) was found. Dancing sign was present in 5/198 (2.5%) dogs. Postural tremors were reported in 10/198 (5.1%) dogs. Worsening of tremors with excitement or noise was reported in 19/198 (9.6%) dogs. Ceasing of tremors while walking was reported in 7/198 (3.5%) dogs. Assessment of tremor presence during general anesthesia was reported in 9/198 (4.5%) dogs; in 6/9, tremors were absent, and in 3/9, tremors were present during general anesthesia. Assessment of tremor presence during sleep was performed in 17/198 (8.6%) dogs; in 13/17, tremors were absent, and in 4/17, tremors were present during sleep. Weight‐bearing lifting limb test (WEBLT) was performed in 6/198 (3%) dogs; this evaluation showed no change in tremor intensity in 3/6 dogs, decreased tremors in 2/6, and cessation of tremors in 1/6 dog. Tremor phenotypes for the most common diagnoses (*n* > 4) in dogs with generalized tremors are presented in Table 3.

**VIDEO 1:** Video compilation of generalized tremors in dogs. Video content can be viewed at https://onlinelibrary.wiley.com/doi/10.1111/jvim.70062

**TABLE 3:** Tremor phenotype in the most common diagnoses (*n* > 4) in dogs with generalized tremors.

Tremor phenotype	Intoxication (*n* = 91)	IGTS (*n* = 49)	Hypocalcemia (*n* = 13)	MUO (*n* = 9)	Hypoglycemia (*n* = 6)	Hypercalcemia (*n* = 5)	Degenerative encephalopathies (*n* = 5)
Periodicity							
Continuous	87/91 (95.6%)	46/49 (93.9%)	10/13 (76.9%)	6/9 (66.7%)	1/6 (16.7%)	5/5 (100%)	4/5 (80%)
Episodic	4/91 (4.4%)	3/49 (6.1%)	3/13 (23.1%)	3/9 (33.3%)	5/6 (83.3%)	0/5 (0)	1/5 (20%)
Additional features (percentages based on the total population reports)							
Deteriorating upon excitement or noise	1/19 (5.2%)	12/19 (63.2%)	0/19 (0)	4/19 (21%)	0/19 (0)	0/19 (0)	0/19 (0)
Postural tremor	0/10 (0)	6/10 (60%)	0/10 (0)	0/10 (0)	0/10 (0)	0/10 (0)	0/10 (0)
Cease while walking	1/7 (14.2%)	2/7 (28.6%)	0/7 (0)	0/7 (0)	0/7 (0)	0/7 (0)	0/7 (0)
Intention head tremor	0/8 (0)	6/8 (75%)	0/8 (0)	0/8 (0)	0/8 (0)	0/8 (0)	0/8 (0)
Palpebral tremor	1/7 (14.2%)	5/7 (71.4%)	0/7 (0)	1/7 (14.3%)	0/7 (0)	0/7 (0)	0/7 (0)
Mandibular tremor	0/5 (0)	3/5 (60%)	0/7 (0)	0/7 (0)	0/7 (0)	0/5 (0)	0/5 (0)
Dancing sign	0/5 (0)	3/5 (60%)	0/5 (0)	1/5 (20%)	0/5 (0)	0/5 (0)	0/5 (0)
Weight‐bearing lifting limb test (WEBLT) (*n* = 6)^a^							
No change	—	3	—	—	—	—	—
Decreased tremors	—	—	—	—	—	—	—
Ceased tremors	—	1	—	—	—	—	—
Assessment during general anesthesia (*n* = 9)							
Present tremors	2	—	—	—	—	1	—
Absent tremors	2	4	—	—	—	—	—
Assessment during sleep (*n* = 17)							
Present tremors	—	4	—	—	—	—	1
Absent tremors	—	11	—	—	—	—	1

Abbreviations: IGTS, idiopathic generalized tremor syndrome; MUO, meningoencephalomyelitis of unknown origin.

^a^ 2/6 dogs had tremors decreasing upon WEBLT and a diagnosis of primary orthostatic tremor.

### 3.4 Statistical Analysis

In the univariable analysis, clinical variables of dogs with intoxication were compared to dogs with IGTS. Dogs with IGTS were female (*p* = 0.002), younger (*p* = 0.002) and had an acute progressive often lateralizing presentation (*p* < 0.001 for all three features). Dogs with intoxication were male (*p* = 0.002), young and middle‐aged (*p* = 0.002) and had a hyperacute nonprogressive symmetric presentation (*p* < 0.001 for all three features). Behavioral changes (*p* = 0.02) and hypersalivation (*p* = 0.04) were more common presenting complaints in dogs with intoxication compared with dogs with IGTS, where hyporexia was more common in IGTS (*p* < 0.001). Dogs diagnosed with intoxication had only tremors (45%) as a neurological finding compared with dogs diagnosed with IGTS (22%; *p* = 0.01). Dogs with intoxication were more likely to manifest abnormal mentation (*p* = 0.01), bilateral mydriasis (*p* = 0.02) or generalized hyperesthesia (*p* = 0.002) compared with dogs with IGTS. Dogs with IGTS were more likely to manifest titubation (*p* < 0.001), head tilt (*p* < 0.001), ataxia of all limbs (*p* < 0.001) or cranial nerve deficits (*p* < 0.001) such as menace response deficits (*p* < 0.001), nystagmus (*p* = 0.003) or opsoclonus (*p* < 0.001) compared with dogs diagnosed with intoxication. Neuroanatomical localization was more likely to be multifocal in intoxication (*p* < 0.001) and vestibulocerebellar in IGTS (*p* < 0.001). Tremors were likely to have additional intentional features in IGTS (*p* < 0.001), whereas additional mandibular (*p* = 0.04) or palpebral (*p* = 0.01) tremor or dancing sign (*p* = 0.04) were more likely to be manifested in IGTS. Improvement within 48 h from the onset of signs without corticosteroid administration occurred exclusively in dogs with intoxication (*p* < 0.001). These dogs received supportive treatment, including IV fluid treatment, activated charcoal, intralipid emulsion, or a combination of them. Multivariable analysis was performed on all variables that reached significance in univariable analysis, but no variables reached statistical significance.

## 4 Discussion

Generalized tremors can be associated with a variety of diseases in dogs, but intoxication and IGTS were most common in our study. In our study, diseases associated with generalized tremors and their frequency were described. Although clinical differentiation between the two most common causes of generalized tremors can be challenging, some historical and clinical features can aid in recognizing the most likely differential diagnosis. This information can assist clinicians in selecting appropriate diagnostic tests and treatment strategies.

The prevalence of dogs presented to primary veterinary practices in the United Kingdom (UK) diagnosed with intoxications or immune‐mediated diseases is low (1.3% and 1%, respectively) [19], and the prevalence of tremors in dogs has not yet been quantified. Anecdotally, generalized tremors are not considered a rare presenting complaint in dogs. No study to date has described the prevalence of underlying diseases associated with generalized tremors in a larger population of dogs. Intoxication appeared to be the most common disease associated with generalized tremor in our study, followed by IGTS. The most common cause of intoxication was unknown, followed by mycotoxins and metaldehyde. Our results are in agreement with the results of a previous study that also considered intoxication and IGTS to be the most common causes of generalized tremors in dogs [3]. In that study, IGTS was the most common cause followed by mycotoxin intoxication [3]. Other common causes of generalized tremors in our study included hypocalcemia, MUO, hypoglycemia, hypercalcemia, and degenerative encephalopathies.

### 4.1 Comparison of Intoxications and IGTS

Dogs with intoxication were usually young or middle‐aged and had a hyperacute nonprogressive symmetric presentation, whereas dogs with IGTS were young with an acute progressive often lateralizing presentation. Intoxication traditionally is hyperacute, with clinical signs usually occurring within 24 h of the potential exposure to the toxic agent [13, 20, 21]. In our study, intoxication usually was diagnosed in young or middle‐aged male dogs in comparison with IGTS. In previous studies, no male overrepresentation was evident in dogs with intoxication, but most dogs were young or middle‐aged [13, 20, 21]. This finding may be explained by the curiosity of younger dogs that may lead them to scavenge more and therefore be more vulnerable to ingesting a potential intoxicant, similar to what has been reported in *Angiostrongylus vasorum* infection where young dogs are more prone to scavenge snails and slugs [22]. The presentation of dogs with IGTS differed from that of dogs with intoxication, because the former are presented with an acute progressive course, whereas the latter present in a more hyperacute nonprogressive fashion with tremors occasionally being the only neurological finding. Thus, the onset of signs in IGTS occurred > 24 h from the time of presentation, which starts with insidious hyporexia and lethargy that progresses to generalized tremors often accompanied by other vestibulocerebellar signs [3, 18, 23, 24, 25]. Dogs with IGTS were young at the age of onset of signs, with a median age of 2 years. Idiopathic generalized tremor syndrome is an immune‐mediated disease of young dogs with reported median ages at onset of signs from 9.6 months to 2 years of age [18, 24, 25]. To date, the terminology of IGTS has been inconsistent, and possibly idiopathic may be a misnomer because there is evidence of immune‐mediated etiology including histopathology consistent with inflammation in few cases [11], CSF analysis consistent with lymphocytic pleocytosis in some cases, response to immunosuppressive doses of corticosteroids in all cases [18, 23, 24], and recent evidence of auto‐antibodies in a small cohort of dogs with severe clinical signs [25]. Additionally, dogs with IGTS may have gastrointestinal signs before development of neurological signs [18]. Idiopathic generalized tremor syndrome has been reported to start with hyporexia and lethargy [18, 24], and 22% of dogs in our study were hyporexic and lethargic. In our study, hyporexia was most likely to occur in dogs with IGTS, which is in agreement with the literature [18, 24]. Hyporexia may be related to nausea associated with severe impairment of the vestibular system, as indicated by the presence of vestibulocerebellar signs. Additionally, dogs with IGTS were most likely to have accompanying vestibulocerebellar signs such as titubation, head tilt, ataxia, menace response deficits, nystagmus or opsoclonus, as reported previously [18, 24]. Some of these signs (e.g., opsoclonus) may not have been consistently recorded in the medical records in both diagnoses, especially in dogs initially examined by non‐neurologists, and therefore results should be interpreted cautiously. On the other hand, our study showed that tremors as the sole neurological finding, behavioral changes, bilateral mydriasis, hypersalivation, abnormal mentation, and general hyperesthesia were associated mostly with a diagnosis of intoxication. This observation is in agreement with the fact that most neurointoxications cause diffuse central nervous system disease affecting multiple regions of the brain [26]. Finally, improvement within 48 h from onset of signs without corticosteroid administration occurred exclusively in dogs with intoxication, which may help direct the clinician towards a more appropriate differential diagnosis during the initial monitoring phase of hospitalized dogs.

Tremor in dogs with IGTS had additional intentional head tremor features in our study, likely because of cerebellar involvement in the disease process [11]. Additional focal tremor such as palpebral tremor, mandibular tremor or opsoclonus was most likely to occur in IGTS, as reported previously [18]. Interestingly, dancing sign was observed in some dogs with IGTS. This sign has been observed in dogs and humans diagnosed with orthostatic tremor of high frequency or intensity, and it has been attributed to relief from the feeling of tremor by elimination of weight‐bearing [10]. Tremor in IGTS represents kinetic tremor. Despite the fact that IGTS is not a postural tremor, some dogs may obtain relief by lifting the limb and trying to minimize unsteadiness. On the other hand, tremors associated with intoxication do not represent actual tremors because they have irregular rhythm and are usually consistent with generalized fasciculations caused by peripheral nerve hyperexcitability (resembling subcutaneous vermicular movements) [2], or epileptic or nonepileptic myoclonus (when the involuntary movement causes movement of a part of the body) [27] associated with the intoxication. Although our study had limited data on tremors during general anesthesia, usually intoxication‐related tremors persisted under general anesthesia in contrast to IGTS tremors. This difference may be explained because their origin may be peripheral nerve hyperexcitability, which can persist during anesthesia [2].

Our study suggests a reconsideration in the diagnostic approach to generalized tremors in dogs. Time is important because the severity of tremors can increase in cases of IGTS, whereas in cases of intoxication increasing severity usually does not occur. Additionally, a basic clinicopathological evaluation for a dog presenting with generalized tremors should include CBC, serum biochemistry (including glucose and electrolytes, including ionized calcium concentration), blood ammonia concentration and bile acid stimulation test. In humans, a genetic panel is obtained commonly in patients with suspected degenerative disease and movement disorders [28]. Genetic testing is not yet common for dogs. However, in dogs with generalized tremors, especially breeds with specific diseases associated with tremors, genetic testing should be considered if commercially available. For instance, in Springer Spaniel (myelin proteolipid protein 1 [PLP1] gene) or Weimaraner (folliculin‐interacting protein 2 [FNIP2] gene) puppies with tremors, genetic tests for hypomyelinating disorders should be considered [29, 30].

Our study had some limitations, including its retrospective nature, lack of video documentation for tremor type in each case, lack of detailed tremor analysis for each case, and lack of electromyography in the awake state for all cases.

In conclusion, several diseases were associated with generalized tremors in dogs, but intoxications and IGTS were most common. Clinically, a hyperacute nonprogressive symmetric presentation occasionally with tremors as the only neurological sign may lead to a diagnosis of intoxication. In contrast, an acute progressive often lateralizing presentation of vestibulocerebellar signs along with generalized tremors that do not improve without corticosteroids in the first 48 h warrants a suspected diagnosis of IGTS. This information may assist the clinician in formulating a differential diagnoses list and instituting appropriate diagnostic and treatment plans.

## Disclosure

Authors declare no off‐label use of antimicrobials.

## Ethics Statement

Authors declare no Institutional Animal Care and Use Committee or other approval was needed. Authors declare human ethics approval was not needed.

## Conflicts of Interest

The authors declare no conflicts of interest.

## Supporting information


**Data S1.** Supporting Information.
