# Supplementary material for: Generalized Tremors in Dogs: 198 Cases (2003–2023)
Source: J Vet Intern Med. 2025 Mar 28;39(3):e70062. doi: 10.1111/jvim.70062 (PMC11951301; doi:10.1111/jvim.70062)
Supplement: Supplementary file 1 — Data S1. Supporting Information. [file JVIM-39-e70062-s001.docx]

**Supplementary material**

**S1**. Causes of metabolic diseases causing generalized tremor in dogs

| **Metabolic abnormality (n, %)** | **Diagnosis, n (%)** |
| --- | --- |
| Ionized hypocalcemia (13/33; 39.4%) | Eclampsia, 4/13 (30.8) |
|  | Chronic kidney disease, 3/13 (23) |
|  | Primary hypoparathyroidism, 2/13 (15.4) |
|  | Respiratory alkalosis in pulmonary carcinoma, 1/13 (7.7) |
|  | Vitamin D deficiency, 1/13 (7.7) |
|  | Pyelonephritis, 1/13 (7.7) |
|  | Acute kidney injury, 1/13 (7.7) |
| Hypoglycemia (6/33; 18.2%) | Insulinoma, 4/6 (66.7) |
|  | Insulin overdose in diabetes mellitus, 1/6 (16.7) |
|  | Severe hypoglycemia in congenital extrahepatic portosystemic shunts, 1/6 (16.7) |
| Ionized hypercalcemia (5/33; 15.2%) | Paraneoplastic - Lymphoma, 2/5 (40) |
|  | Paraneoplastic - Intestinal sarcoma, 1/5 (20) |
|  | Paraneoplastic - Multiple myeloma, 1/5 (20) |
|  | Paraneoplastic - Suspected bone neoplasia, 1/5 (20) |
| Hepatic encephalopathy - portosystemic shunts (PSS) (4/33; 12.1%) | Extra-hepatic PSS, 3/4 (75)   - Hyperammonemia, 1/1 - Increased BAST, 4/4 - PANS, 1/4 |
|  | Intra-hepatic PSS, 1/4 (25)   - Hyperammonemia, 1/1 - Increased BAST, 1/1 |
| Hyperammonemia (1/33; 3%) | Acute kidney disease, diabetic ketoacidosis, acute kidney injury, hepatopathy |
| Hypernatremia (1/33; 3%) | Pyometra |
| Hypoadrenocorticism (1/33; 3%) | - |
| Diabetic polyneuropathy (1/33; 3%) | Diabetes mellitus |
| Suspected hypercatecholemia (1/33; 3%) | Left adrenal pheochromocytoma |

PSS: Portosystemic shunts; DKA: diabetic ketoacidosis; BAST: bile acid stimulation test

**S2**. Breeds of dogs manifesting generalized tremors

| **Breeds** | **n (%)** |
| --- | --- |
| Crossbreed | 49/198 (24.7) |
| Labrador | 22/198 (11.1) |
| Jack Russell Terrier | 19/198 (9.6) |
| West Highland White Terrier | 14/198 (7.1) |
| Cocker Spaniel | 7/198 (3.5) |
| Dachshund | 6/198 (3) |
| English Springer Spaniel | 5/198 (2.5) |
| Schnauzer | 5/198 (2.5) |
| Chihuahua | 4/198 (2) |
| Staffordshire Bull Terrier | 4/198 (2) |
| Bichon Frise | 3/198 (1.5) |
| Yorkshire Terrier | 3/198 (1.5) |
| Border Collie | 2/198 (1) |
| Cockapoo | 2/198 (1) |
| Dobermann | 2/198 (1) |
| German Short-haired pointer | 2/198 (1) |
| Giant Schnauzer | 2/198 (1) |
| Miniature Pinscher | 2/198 (1) |
| Miniature Poodle | 2/198 (1) |
| Miniature Schnauzer | 2/198 (1) |
| Saluki | 2/198 (1) |
| Weimaraner | 2/198 (1) |
| Whippet | 2/198 (1) |
| Welsh Springer Spaniel | 2/198 (1) |
| Australian Labradoodle | 2/198 (1) |
| Basset Hound | 1/198 (0.5) |
| Beagle | 1/198 (0.5) |
| Border Terrier | 1/198 (0.5) |
| Boston Terrier | 1/198 (0.5) |
| Boxer | 1/198 (0.5) |
| Cairn Terrier | 1/198 (0.5) |
| Collie | 1/198 (0.5) |
| Coton De Tulear | 1/198 (0.5) |
| Dalmatian | 1/198 (0.5) |
| English Bulldog | 1/198 (0.5) |
| French Bulldog | 1/198 (0.5) |
| Fox Terrier | 1/198 (0.5) |
| German long-haired pointer | 1/198 (0.5) |
| German Spitz | 1/198 (0.5) |
| Great Dane | 1/198 (0.5) |
| Greyhound | 1/198 (0.5) |
| Italian Greyhound | 1/198 (0.5) |
| Koikerjondje | 1/198 (0.5) |
| Lakeland Terrier | 1/198 (0.5) |
| Lhasa Apso | 1/198 (0.5) |
| Lurcher | 1/198 (0.5) |
| Manchester Terrier | 1/198 (0.5) |
| Pearson Russel Terrier | 1/198 (0.5) |
| Petit Brabancon | 1/198 (0.5) |
| Pomeranian | 1/198 (0.5) |
| Poodle | 1/198 (0.5) |
| Pug | 1/198 (0.5) |
| Scottish Terrier | 1/198 (0.5) |
| Sealy Dale Terrier | 1/198 (0.5) |
| Shih Tzu | 1/198 (0.5) |
| Slovakian rough haired Pointer | 1/198 (0.5) |
| Tibetan Terrier | 1/198 (0.5) |
| Hungarian Vizsla | 1/198 (0.5) |
